# New *Lactiplantibacillus plantarum* and *Lacticaseibacillus rhamnosus* strains: well tolerated and improve infant microbiota

**DOI:** 10.1038/s41390-021-01678-1

**Published:** 2021-08-24

**Authors:** Gunilla Önning, Ragnhild Palm, Caroline Linninge, Niklas Larsson

**Affiliations:** 1 Probi AB, Lund, Sweden; 2 Biomedical Nutrition, Pure and Applied Biochemistry, Center for Applied Life Sciences, Lund University, Lund, Sweden; 3 Medical Department for Children and Adolescents, Landskrona Hospital, Landskrona, Sweden; 4 Department of Food Technology, Engineering and Nutrition, Lund University, Lund, Sweden

## Abstract

**Background:**

Different microorganisms from the environment will begin to colonise the infant during and immediately after the delivery. It could be advantageous to influence the microbiome early on by giving infants probiotic bacteria. The aim of the study was to investigate the tolerance of two probiotic lactobacilli in infants. The effect on the microbiota was also followed.

**Methods:**

Thirty-six healthy infants, aged 4–83 days at the start of the study, were given a daily supplementation of probiotics (*Lactiplantibacillus plantarum* HEAL9 and *Lacticaseibacillus rhamnosus* 271, 10^9^ CFU (colony-forming units)) or placebo for 8 weeks. Adverse events, growth parameters, the faecal microbiome and intestinal performance were followed.

**Results:**

No differences between the groups in growth parameters, adverse events and intestinal performance were observed. The faecal levels of *L. plantarum*, *L. rhamnosus* and lactobacilli increased after the intake of probiotics and were significantly higher compared with the placebo group after 4 and 8 weeks of intake. The faecal microbial diversity was similar in the two groups at the end of the study.

**Conclusions:**

The intervention with the probiotic formulation was well tolerated and increased the level of lactobacilli in the intestine. The developed probiotic formulation will be further evaluated for clinical efficacy in infants.

**Impact:**

- New data for the development of the gut function and the microbiome in breastfed and/or formula-fed young infants over time and the effect of adding two probiotic strains are presented.
- *Lactiplantibacillus* *plantarum* is a species that seldom has been analysed in infants, but it could be detected in 25% of the subjects before administration (mean age 41 days).
- *Lactiplantibacillus* *plantarum* and *L. rhamnosus* establish well in the intestine of infants and are well tolerated.
- The microbiota was positively affected by the intake of probiotics.

## Background

Different microorganisms from the environment will begin to colonise the infant during and immediately after delivery. The earliest encounters of bacteria the infant is exposed to include the birth canal, which is dominated by lactobacilli, the maternal faecal microbiota, in children born vaginally, skin and breast-milk bacteria, as well as the bacterial environment in the hospital or surrounding environment. During the first year of life, diet plays an important role in shaping the microbiota composition as the infant transitions from breast-feeding or bottle-feeding to other more complex foods^1^.

According to the currently adopted definition, probiotics are: ‘Live microorganisms that, when administered in adequate amounts, confer a health benefit on the host’^2^. Different studies have suggested that it could be advantageous to influence the microbiome early on by giving infants probiotic bacteria. Giving probiotics to children with colic can be valuable since these children seem to be less colonised by the genus *Lactobacillus*^3^ and also seem to have a less diverse faecal microbiota^4^. The intake of probiotics may also reduce the risk of developing coeliac disease and other autoimmune diseases, as well as of gastrointestinal infections^5,6^.

Giving probiotics to healthy infants does not raise concerns regarding the growth and adverse effects according to the ESPGHAN committee on nutrition^5^. However, the committee also concludes that the safety of new probiotic bacterial strains cannot be extrapolated from other already investigated strains^5^. Thus, the objective of the study was to investigate the tolerance, survivability and activity of two bacterial strains that never have been given to infants before (*Lacticaseibacillus rhamnosus* 271 and *Lactiplantibacillus plantarum* HEAL9). The strains were carefully selected based on previous studies, showing both intestinal survival and establishment in adults,^7–9^ and different strain-specific immune-strengthening properties,^10,11^ which provides a scientific basis for combining the two strains to increase the possibility of a health benefit in infants. *Lacticaseibacillus rhamnosus* 271 and *L. plantarum* HEAL9 belong to species commonly found on oral and rectal mucosa of a healthy human^12^. The occurrence of *L. rhamnosus* is mostly reported in association with consumption of dairy products, while *L. plantarum* is associated with the intake of lactic acid-fermented foods of plant origin like brined olives, sauerkraut, salted gherkins and sourdough^13^. The occurrence of lactobacilli in the microbiota of Swedish breastfed infants (*n* = 112) has been investigated previously^14^. Lactobacilli were isolated from 21% of the stool samples from 1-week-old infants, and at 8 weeks of age, the incidence had increased to 34%. *Lacticaseibacillus rhamnosus* was one of the most commonly isolated species, while *L. plantarum* was only isolated from one child.

Strains of *L. rhamnosus* have for a long time been used as probiotics for infants and children in a wide range of different probiotic products, marketed in many countries. One of the most studied strains is *L. rhamnosus* GG, which is well tolerated and safe for infants and children^15,16^. The specific *L. rhamnosus* strain investigated in this study, *L. rhamnosus* 271 (DSM (Deutsche Sammlung von Mikroorganismen) 6594), was originally isolated from colonic mucosa of a healthy human^17^ and has been studied in several human trials in adult subjects without any reported adverse events^7,9,11^. *Lacticaseibacillus rhamnosus* 271 has, in a pilot trial, been shown to down-regulate the cell-mediated immunity in adults^11^ and in animal studies to reduce bacterial translocation^18,19^, making it interesting to include in a product for infants. Different strains of *L. plantarum* have also been widely used as probiotics and one well-known strain is *L. plantarum* 299v, which has safely been given to immune-compromised children with human immunodeficiency virus^20^ and in high doses to healthy children between the ages of 6 months and 3 years (1 × 10^11^ colony-forming units (CFU)/day)^21^. *Lactiplantibacillus plantarum* HEAL9 (DSM 15312) investigated in this study was isolated from the colonic mucosa of a healthy human and found in faeces and vagina after oral administration in adults without any adverse events^8,22^. One further basis for the selection of this strain was that it has been shown to influence the immune defense by decreasing the risk of acquiring common cold infections in adults (combined with *L. paracasei* 8700:2 in a ratio of 1:1)^8,23^.

The primary objective of this parallel, placebo-controlled, double-blind, randomised pilot study was to evaluate the tolerance of *L. plantarum* HEAL9 and *L. rhamnosus* 271 in healthy infants. A probiotic bacterial strain should be able to reach the gastrointestinal tract, and this was included as a secondary objective together with an investigation of the impact the probiotic formulation could have on other bacterial groups in faeces. The total dose in the study was 10^9^ CFU/day, and due to previous good experience from combining *L. plantarum* HEAL9 with another bacterial strain in a 1:1 ratio, the same ratio between the strains was used in the present study.

## Methods

### Study design

The study was randomised, double-blind and placebo-controlled with two parallel arms. After a run-in period of 1 week (day 1–7), subjects were divided into one probiotic arm and one placebo arm for an intervention period of 8 weeks (days 8–63). The subjects consumed either a probiotic formulation (10^9^ CFU/day) or a placebo product. Visits were made before the run-in period (visit 1), after the run-in period (visit 2) and after 4 and 8 weeks of intervention (visit 3 and 4). The study was performed between April and December 2011 in child healthcare centres in the area of Landskrona, Sweden. Permission to carry out the study was given by the Regional Ethical Review Board in Lund (2011/41). The study was carried out following the principles in the World Medical Association Declaration of Helsinki, Good Clinical Practice. Verbal information was given to the child’s parents or legal guardians at visit 1 and they gave their written consent to participate before the child was included in the study. Participation was completely voluntary, and the child could discontinue the study at any time without explanation. The study was registered at ClinicalTrials.gov (NCT03925558).

### Study population

Children were recruited during their regular visits to the healthcare centre. Health professionals informed about the inclusion and exclusion criteria, preselected children and handed out written information about the study to the parents. A study nurse contacted the parents later and booked those willing to participate for a first visit. The same study nurse was responsible for the contact with the children and parents throughout the study. Inclusion criteria were full-term, healthy infants aged 3–95 days. Exclusion criteria were prematurity (<36 weeks gestation), low birth weight (<2500 g), congenital anomalies, chronic disease, failure to thrive (weight loss of >2 percentiles), allergy or atopic disease and exposure to antibiotics. Included children were randomised using a computer-generated list with a block size of eight to either probiotic or a placebo product (1:1) at visit 1 and each subject was given a number. The randomisation list was generated by an independent Clinical Research Organisation in Uppsala, Sweden (Good Food Practice) and information regarding the number and subject was kept at Probi AB, separately from other data pertaining to the study. Participants and the study team were blinded to the interventions until the completion of the statistical analysis.

### Study product

The test product was a combination (1:1 ratio) of two probiotic strains, *L. rhamnosus* 271 (DSM 6594) and *L. plantarum* HEAL9 (DSM 15312), at a total daily dose of 1 × 10^9^ CFU, SiO_2_ (0.5%), inulin (0.92%) and glucose/fructose/sucrose (0.08%) in an oil excipient (rape seed oil). The placebo product contained the same ingredients except the lactobacilli to mimic the test product in taste and appearance. The study products were dispensed into glass bottles and the daily intake was four drops (0.2 ml) taken in association with a meal. The study products were kept refrigerated and were delivered to the subjects at visits 2 and 3 (one bottle at each visit). The intake of the study product was reported daily in a diary.

From days 1 to 63, the children were not allowed to ingest products containing other probiotic bacteria. The parents were provided with a list of probiotic products that should not be consumed during the study and were asked to register compliance in the diary daily.

### Tolerability follow-up parameters

The primary outcome of the study was a comparison of different growth parameters between the probiotic and the placebo group after 8 weeks of intervention. The child’s weight without clothes or diaper (to the nearest 5 g), length (to the nearest 0.5 cm) and head circumference (to the nearest 0.1 cm) was determined at baseline and after 4 and 8 weeks of intervention during the visits to the healthcare centre. Secondary outcomes were registered daily in a diary by the parents: Information on general health status (occurrence of any illness, healthcare visits for sickness, medication and antibiotic use), gastrointestinal signs and related symptoms (number of stools, stool consistency (on a 1–3 scale; watery, loose-normal, hard), flatulence (on a 1–3 scale; none, moderate, abundant), vomiting /regurgitation (yes or no), abdominal pain and crying time (on a 1–5 scale; none, <1 h, 1–2 h, 2–3 h, >3 h). Adverse events were recorded during the visits to the healthcare centre after 4 and 8 weeks of intake by asking the parents and by checking the diary.

### Sampling of faeces and microbial analysis

Faecal samples were collected from diapers in sterile plastic tubes and stored at <−18 °C. The samples were delivered at the next visit to the healthcare centre and stored at −20 °C for later collection and long-term storage at −80 °C. Samples were taken before the start of the intervention and after 4 and 8 weeks of intake.

The microbiological analysis was done using quantitative real-time polymerase chain reaction (qPCR). The samples were analysed for *L. plantarum*, *L. rhamnosus*, lactobacilli, bifidobacteria, *Escherichia coli*, *Bacteroides–Prevotella–Porphyromonas* and *Clostridium* group XI. DNA was extracted using Nordiag Arrow Stool DNA Kit (Nordiag) according to the manufacturer’s instructions. The amount of faeces collected for DNA extraction was ~0.060 g. The qPCR analysis was performed with a Realplex Mastercycler, Eppendorf.

### Analysis of *L. plantarum* and *L. rhamnosus*

To analyse the level of *L. plantarum* and *L. rhamnosus* in the stool samples, qPCR assays were performed as previously described^24^ after optimisation. In Haarman and Knol,^24^ the selection of primer and probe sequences were done by retrieving 16S–23S intergenic spacer regions of the different *Lactobacillus* species from the GenBank, EMBL and DDBJ databases. Primers and probes were obtained from Applied Biosystems (see Supplementary Table 1). The assays were performed with a 25 μl PCR-amplification mixture containing 12.5 µl Platinum Quantitative PCR SuperMix-UDG (Invitrogen), optimised concentrations of the primers and probes and 5 µl DNA extracted from stool samples. The temperature profile for the amplification consisted of one incubation step for 2 min at 50 °C, denaturation step for 2 min at 95 °C, followed by 40 cycles with 3 s at 95 °C and 30 s at 57 °C (*L. plantarum*) or 56 °C (*L. rhamnosus*). The standard curves for *L. rhamnosus*, *L. plantarum* and total lactobacilli were created by extracting DNA from pure cultures of *L. plantarum* HEAL9 and *L. rhamnosus* 271. All reactions were performed in triplicate.

### Analysis of lactobacilli, *E. coli*, bifidobacteria, *Bacteroides–Prevotella–Porphyromonas* and *Clostridium* group XI

The reaction mixture contained 5 µl of Platinum^®^ SYBR^®^ Green qPCR SuperMix-UDG (Invitrogen) primer at a concentration of 0.5 µM except for *E. coli* where the primer concentration was 1.0 µM (TAG Copenhagen A/S). To each reaction 2 µl of template DNA, extracted from stool samples, was added. Initially, the thermal cycling consisted of one incubation step at 50 °C for 2 min and a denaturation step at 95 °C for 2 min. This was followed by 40 cycles with denaturation at 95 °C for 15 s, annealing for 30 s and one elongation step at 72 °C for 30 s. At the end of each reaction, a melting-curve analysis was performed by slowly heating the reaction mixture from 60 to 95 °C. To obtain a standard curve, purified bacterial DNA was obtained from CCUG (Culture Collection University of Gothenburg). DNA from the following bacterial strains was used: *E. coli* CCUG 24T, *Bacteroides fragilis* CCUG 4856T, *Clostridium lituseburense* CCUG 18920T and *Bifidobacterium adolescentis* CCUG 17359T. All reactions were performed in triplicate. The primers used and PCR conditions are shown in Supplementary Table 2.

### Analysis of the microbial diversity

Terminal restriction fragment length polymorphism (T-RFLP) analysis was used to evaluate the diversity. The 16S ribosomal RNA genes were amplified with universal primers as previously described.^25^ Amplification was carried out in an Eppendorf Mastercycler for 25 cycles and PCR products were verified and purified as described by Karlsson et al.,^25^ with the modification that 0.4 µl FAM-ENV1 primer was used. T-RFLP was performed and analysed as described elsewhere^25^. Thresholds for internal standard and T-RFs were set to 20 and 25 fluorescence units, respectively. Microbial diversity was evaluated by counting the number of T-RF in the T-RFLP profile of each sample. Furthermore, the relative abundance of each T-RF within a given T-RFLP pattern was calculated as the peak area of the respective T-RF divided by the total peak area of all T-RFs, in the given T-RFLP pattern, detected within a fragment length of 40 to 580 base pairs (bp). Shannon (*H*′) and Simpson (*D*) indices were calculated by using the equations: *H*´ =−Σ*p*_*i*_ ln *p*_*i*_ and 1 − *D*, where *D* = Σ*p*_*i*_^2^, where *p*_*i*_ is the relative abundance of the *i*th peak in the community^26^. The difference in diversity was obtained by the following calculation: diversity index after treatment – diversity index before treatment = change in bacterial diversity.

### Statistical evaluation

The number of subjects included in the study was based on an earlier tolerance study^27^. The data from all randomised subjects were analysed on an intention-to-treat basis. Descriptive statistics (number of observations, minimum and maximum values, standard deviation or median) were used. The different measured numerical data were compared between the groups using the independent *t* test (tolerance data) and the Mann–Whitney test (microbiome data). Changes over time within a group were evaluated with the Wilcoxon signed-rank test. For comparisons of categorical data, Fisher’s exact test was used. The level of significance was set at *p* < 0.05. The statistical analyses were performed by Good Food Practice, in Uppsala, Sweden, using SPSS version 15.0.

To reveal differences in diversity before and after probiotic consumption, multivariate data analysis with orthogonal partial least squares to latent structures discriminant analysis (OPLS-DA) was performed using SIMCA 13.0 (Umetrics AB, Umeå, Sweden)^28,29^. The individual diversity changes in the probiotic group were also compared with the changes within the placebo group using Mann–Whitney rank-sum test (SigmaStat 3.1, Systat Software, Point Richmond, CA) and *p* values <0.05 were considered statistically significant.

## Results

### Study population

**Fig. 1:**
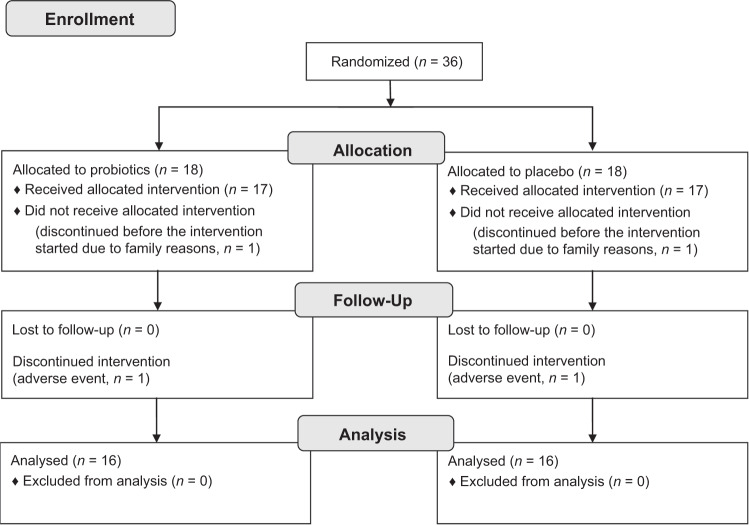
CONSORT diagram. Flow of subjects through each stage of the study.

Thirty-six children were included in the study and 32 completed the study (16 in each group). Two subjects withdrew from the study before administration of study product due to family reasons (one in the placebo group and one in the probiotic group), one subject terminated in the probiotic group due to a serious adverse event unlikely related to the intake of study product (detection of multiple ventricle septum defects) and one terminated in the placebo group due to an adverse event (gastroenteritis) (Fig. 1). Baseline characteristics per treatment group are presented in Table 1. The children were between 4 and 83 days old (mean age 41 days) at inclusion. Three children were born with caesarean section (8%), the majority of the children were breastfed at inclusion (84%) and four of the children had been given probiotics previously (11%). No differences in baseline characteristics were found except for breast-feeding at inclusion where all children in the probiotic group were breastfed compared to 69% in the placebo group (*p* = 0.029). At the end of the study, most of the children were still breastfed (81%).

**Table 1:** Baseline characteristics per treatment group^a^.

	Probiotics (*n* = 18)	Placebo (*n* = 18)
Gender male/female	6/12	9/9
Gestational age (weeks), mean (SD)	40.0 (1.2)	40.1 (1.3)
Birth weight (g), mean (SD)	3556 (591)	3767 (436)
Length at birth (cm), mean (SD)	52 (2)	52 (2)
Head circumference at birth (cm), mean (SD)	35.3 (1.9)	35.7 (1.2)
Born with caesarean section, *n* (%)	1 (6%)	2 (11%)
Mother treated with antibiotics during the pregnancy, *n* (%)	3 (17%)	3 (17%)
Mother eating probiotics in connection with the childbirth, *n* (%)	0 (0%)	1 (6%)
Having family member with allergy, *n* (%)	9 (50%)	6 (33%)
Age at inclusion (days), mean (SD)	42 (14)	41 (18)
Intake of probiotics at inclusion, *n* (%)	1 (6%)	3 (18%)
Exclusively breastfed, *n* (%)^b^	10 (62%)	6 (38%)
Both breastfed and formula-fed, *n* (%)^b^	6 (38%)	5 (31%)
Exclusively formula-fed, *n* (%)^b^	0 (0%)	5 (31%)

^a^There were no significant differences between groups in baseline characteristics except for breast-feeding at inclusion (*p* = 0.029, Fisher’s exact test).

^b^Number of analysed infants: 16 per group.

### Tolerability

The intake of study products started 1 week after inclusion at a mean age of 48 days. The test products were well tolerated and the mean (min–max) compliance was 98% (86–100%) in the probiotic group and 91% (46–100%) in the placebo group. One child in the placebo group was given a probiotic supplement during the run-in week and the first two intervention weeks and another child in the placebo group consumed occasionally an infant formula with probiotics during the study.

Weight, length and head circumference at inclusion and after 4 and 8 weeks of administration are presented in Table 2. The mean weight (SD) at inclusion was 4730 (890) g for the probiotic group and 4920 (780) g for the placebo group and no significant differences in the weight development between the groups were found at weeks 4 and 8. The length was similar in both groups at the start (57 cm) and at the end of the study (63–64 cm) as was head circumference and no significant differences between the groups were found at any time point.

**Table 2:** Weight, length and head circumference at inclusion and after 4 and 8 weeks of administration (mean (SD))^a^.

	Before administration (at inclusion), *n* = 18 per group	After 4 weeks of administration, *n* = 17 per group	After 8 weeks of administration, *n* = 16 per group
Weight (g)			
Probiotics	4725 (890)	5595 (910)	6335 (875)
Placebo	4915 (780)	5885 (900)	6655 (760)
Length (cm)			
Probiotics	56.5 (3.0)	61.0 (2.5)	63.5 (2.5)
Placebo	57.5 (3.0)	61.5 (3.5)	64.5 (2.0)
Head circumference (cm)			
Probiotics	38.2 (1.8)	39.9 (1.8)	41.1 (1.8)
Placebo	38.6 (1.5)	40.3 (1.3)	41.7 (1.2)

^a^There were no significant differences between groups in weight, length and head circumference at any time point.

The mean number of stools decreased during the study in a similar way for both the probiotic and placebo groups, from 1.9 and 2.1 bowel movements per day before administration to 0.8 and 1.0 bowel movements after 8 weeks of administration, respectively (Table 3). The mean stool consistency per day was similar in both groups before administration (loose-normal) and did not change during the study. Most of the children experienced some flatulence (94%) and stomach pain (75%) during the week before administration. After 8 weeks of administration, the corresponding figures were lower, 70% and 37%, respectively; however with no difference between the groups. Eighty-four percent of the children were crying at baseline, and after 8 weeks of administration, this number was reduced to 73%, but with no difference between groups. There were two children in the study that suffered from colic (cried more than 3 h during at least 3 days in 1 week), one in each intervention group. The mean frequency of vomiting/regurgitation was the same in both groups and did not change during the study.

**Table 3:** Intestinal function the week before administration and during weeks 4 and 8 of administration (mean per day (min–max))^a^.

	Before administration	After 4 weeks of administration	After 8 weeks of administration
Stool frequency			
Probiotics	1.9 (0.3–4.7)	1.5 (0.6–3.1)	0.8 (0.3–1.7)
Placebo	2.1 (0.3–10.0)	1.2 (0.1–5.3)	1.0 (0.2–2.3)
Stool consistency, 1 = watery, 3 = hard			
Probiotics	1.9 (1.0–2.0)	1.9 (1.0–2.5)	1.8 (1.0–2.3)
Placebo	1.9 (1.0–2.5)	2.0 (1.0–3.0)	2.0 (1.8–2.5)
Degree of flatulence, 1 = none, 3 = abundant			
Probiotics	1.9 (1.0–3.0)	1.9 (1.0–2.7)	1.8 (1.0–3.0)
Placebo	2.0 (1.2–2.8)	1.8 (1.0–2.4)	1.6 (1.0–2.7)
Vomiting/regurgitation, 1 = yes, 2 = no			
Probiotics	1.4 (1.0–2.0)	1.5 (1.0–2.0)	1.5 (1.0–2.0)
Placebo	1.5 (1.0–2.0)	1.5 (1.0–2.0)	1.5 (1.0–2.0)
Abdominal pain, 1 = none, >3 h = 5			
Probiotics	1.5 (1.0–2.7)	1.4 (1.0–2.7)	1.2 (1.0–2.0)
Placebo	1.8 (1.0–3.6)	1.8 (1.0–4.0)	1.4 (1.0–3.5)
Crying time, 1 = none, >3 h = 5			
Probiotics	1.9 (1.0–3.6)	1.8 (1.0–3.3)	1.7 (1.0–3.0)
Placebo	2.0 (1.0–3.4)	1.9 (1.0–4.0)	1.7 (1.0–3.5)

Probiotics (*n* = 16) and placebo (*n* = 14–16).

^a^There were no significant differences between groups at any time point.

### Faecal microbiome

**Fig. 2:**
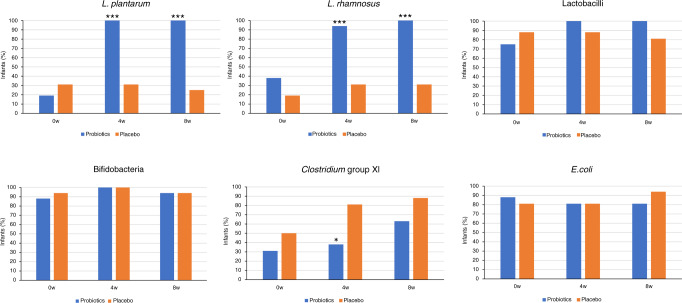
Number of children (%) who had detectable levels of different bacteria in their faeces at the start, and after 4 and 8 weeks administration of probiotics or placebo. *p < 0.05, ***p < 0.001, Fisher’s exact test.

The microbiome was analysed in faeces before the intake of product and after 4 and 8 weeks of intake (Table 4 and Fig. 2).

**Table 4:** Faecal bacterial copies (log10/g) before and after 4 and 8 weeks of administration of probiotics or placebo (median (min–max)).

	Before administration, *n* = 16 per group	After 4 weeks of administration, *n* = 16 per group	After 8 weeks of administration, *n* = 16 per group
*L. plantarum*			
Probiotics	4.5 (<3.7–5.7)	6.4 (4.8–8.1)^a^	6.6 (4.5–7.5)^a^
Placebo	4.5 (<4.3–6.1)	4.5 (<4.2–5.9)	4.4 (<4.1–5.9)^a^
Difference^b^	*p* = 0.239	*p* < 0.001	*p* < 0.001
*L. rhamnosus*			
Probiotics	4.4 (<3.2–8.0)	6.3 (<3.0–7.7)	7.1 (5.1–8.9)
Placebo	4.1 (<3.8–7.9)	3.1 (<2.8–7.2)^a^	4.0 (<3.8–7.4)
Difference^b^	*p* = 0.624	*p* < 0.001	*p* < 0.001
Lactobacilli			
Probiotics	7.1 (<3.7–9.1)	7.8 (5.3–9.1)	7.4 (5.9–9.0)
Placebo	6.3 (<4.3–7.9)	5.6 (<4.4–8.1)	5.5 (<4.3–7.5)^a^
Difference^b^	*p* = 0.407	*p* < 0.001	*p* < 0.001
Bifidobacteria			
Probiotics	10.8 (<5.1–12.2)	10.8 (6.7–12.9)	11.0 (<5.6–12.0)
Placebo	10.8 (<5.9–11.7)	10.7 (9.5–12.2)	10.5 (<5.7–11.6)
Difference^b^	*p* = 0.970	*p* = 0.970	*p* = 0.386
*Bacteroides–Prevotella–Porphyromonas*			
Probiotics	9.9 (<5.5–>11.4)	10.6 (<6.8–>11.4)	10.7 (<6.0–>11.4)
Placebo	8.8 (<6.1–>11.4)	9.8 (<6.2–>11.3)	9.5 (<6.1–>12.0)
Difference^b^	*p* = 0.451	*p* = 0.187	*p* = 0.060
*Clostridium* group XI			
Probiotics	4.8 (<4.0–9.5)	5.2 (<4.5–8.9)	6.7 (<4.4–9.0)^a^
Placebo	5.8 (<4.6–9.0)	7.7 (<4.6–8.8)	7.7 (<4.6–9.0)
Difference^b^	*p* = 0.109	*p* = 0.018	*p* = 0.090
*E. coli*			
Probiotics	10.4 (<5.2–11.7)	10.5 (<5.0–11.8)	10.4 (<5.1–12.3)
Placebo	10.5 (<5.4–11.6)	10.6 (<5.1–11.6)	10.5 (<5.4–11.4)
Difference^b^	*p* = 0.910	*p* = 0.346	p = 0.880

^a^Comparison with before administration within group, Wilcoxon signed-rank test.

^b^Comparison between groups. Mann–Whitney test.

*Lactiplantibacillus plantarum* could be detected in the faeces from 25% of the children before administration. After 4 weeks of the intake of probiotic product, the median level of *L. plantarum* increased significantly (*p* = 0.001) to 6.4 log_10_ copies/g faeces and the bacteria could be detected in all children. No further increase was seen after 8 weeks as the median level of *L. plantarum* was similar (6.6 log_10_ copies/g faeces). *Lactiplantibacillus plantarum* could still be detected in 25% of the children in the placebo group at the end of the study, but the median level was reduced over time and at week 8 it was significantly lower compared with the level before administration and significantly lower than in the faecal samples from the probiotic group at weeks 4 and 8 (*p* < 0.001).

*Lacticaseibacillus rhamnosus* was detected in the faeces from 28% of the children at the start. The median level of *L. rhamnosus* in the probiotic group increased from 4.4 to 6.3 and 7.1 log_10_ copies/g faeces after 4 and 8 weeks of administration, respectively, and at the end of the study, all children in the probiotic group had *L. rhamnosus* in their faeces. The median levels of *L. rhamnosus* in the placebo group were significantly lower after 4 weeks compared to start and significantly lower than in the faecal samples from the probiotic group at week 4 and 8 (*p* < 0.001). *Lacticaseibacillus rhamnosus* was detected in 31% of the children in the placebo group at the end of the study.

The total level of lactobacilli also increased after the intake of the probiotic product and the levels after 4 and 8 weeks were significantly higher than the levels in the placebo group (*p* < 0.001). At the start, 75% of the children in the probiotic group had lactobacilli in their faeces and after 4 and 8 weeks of intake, lactobacilli were detected in the faeces of all children. At the start, 87% of the children in the placebo group had lactobacilli in their faeces and the number was the same throughout the study, with no significant difference compared to the probiotic group.

Bifidobacteria could be detected in the faeces of most of the children at the start (91%) and no significant changes were noticed after 4 and 8 weeks of the intake of probiotic or placebo products. At the start, 84% of the children had detectable levels of *Bacteroides–Prevotella–Porphyromonas* and *E. coli* in the faeces and there were no significant changes detected after the intake of probiotic or placebo product during the study. Before administration, 41% of the children had detectable levels of *Clostridium* group XI bacteria in the faeces with no significant difference between groups (*p* = 0.47). After 4 weeks of administration, significantly more children in the placebo group than in the probiotic group had detectable levels of faecal *Clostridium* group XI bacteria (*p* = 0.029). At the end of the study, 62% of the children in the probiotic group had detectable levels of *Clostridium* group XI bacteria compared to 87% in the placebo group; however, the difference was not significant (*p* = 0.22).

The faecal microbial diversity was not significantly changed after 8 weeks of administration of the probiotic product. Neither the number of T-RF nor the Shannon or the Simpson diversity indices indicated any significant change of the dominating gut microbiota in the infants after probiotic administration when compared to the placebo group (Table 5). Results of multivariate data analysis with OPLS-DA depict a difference in the dominating gut microbiota of infants administered probiotics since samples before administration were separated from samples after probiotic administration (Supplementary Figure 1).

**Table 5:** Faecal microbial diversity, assessed by T-RFLP, before and after 8 weeks of administration of probiotics or placebo (median (min–max))^a,b^.

	Before administration, *n* = 15 per group	After 8 weeks of administration, *n* = 15 per group	Change (after–before), *n* = 15 per group
Shannon			
Probiotics	1.579 (0.465–2.408)	1.670 (0.635–2.101)	–0.0595 (–1.104 to 1.113)
Placebo	1.484 (0.309–2.280)	1.612 (0.288–2.332)	0.085 (–1.310 to 1.118)
Simpson			
Probiotics	0.704 (0.190–0.857)	0.719 (0.278–0.822)	–0.029 (–0.306 to 0.542)
Placebo	0.658 (0.111–0.833)	0.618 (0.098–0.858)	0.034 (–0.560 to 0.458)
No T-RF			
Probiotics	12 (5–24)	12 (6–19)	–1 (–14 to 14)
Placebo	12 (6–24)	11 (8–24)	–1 (–11 to 15)

^a^The diversity could not be evaluated due to a low DNA concentration in samples from one child per group.

^b^There were no significant differences at any time point or in the change (after–before) between groups.

### Adverse events

In total, 14 adverse events were reported during the study (Table 6). Besides the reported serious adverse event in the probiotic group (detection of multiple ventricle septum defects, unlikely related to the intake of study product), flatulence was reported in two children, fever in one child and eczema in one child. In the placebo group, nine adverse events were reported for seven children (constipation, gastroenteritis, fever and upper respiratory infections). None of the infants in the probiotic group experienced an upper respiratory tract infection, while four out of 16 infants (25%) in the placebo group suffered from an infection during the study (*p* = 0.10).

**Table 6:** Number of adverse events (including all children who were administered study product).

Adverse event	Probiotics (*n* = 17)	Placebo (*n* = 17)
Flatulence	2	0
Constipation	0	2
Gastroenteritis	0	1
Multiple ventricle septum defects	1	0
Fever	1	2
Upper respiratory infection	0	4
Eczema	1	0
Total	5	9

## Discussion

The present study was undertaken to investigate the tolerance of a probiotic formulation containing *L. plantarum* HEAL9 and *L. rhamnosus* 271 in infants. The intake of the study product was well tolerated and did not result in any adverse effects on growth or infant behaviour. Similar results have also been obtained with other probiotic strains of the same dosage. In a study where *Bifidobacterium lactis* BB-12 or *L. reuteri* ATCC 55730 were included in an infant formula, no negative effects on growth parameters or variables of feeding, stooling, crying or irritability was detected, in comparison to the control group after 4 weeks of intake^27^. According to a review, other studies that included probiotics in infant formula had in general also no significant effects on growth^30^.

*Lactiplantibacillus plantarum* could be detected in 25% of the infants before administration when they were 4–83 days old. This is a higher figure compared to an earlier trial in 112 infants from Sweden where *L. plantarum* could only be found in the faeces of a single child at the age of 1 week and 4 weeks, in five children at 6 months (4.5%) and in seven children at 12 months (6%)^14^. The same study also analysed the content of *L. rhamnosus* in faeces and the level was peaking between 2 and 6 months of age when the species could be detected in 21% of the children. In the present study, *L. rhamnosus* was detected in 28% of the children at the start.

The intake of the same dose (5 × 10^9^ CFU) of *L. plantarum* HEAL9 by healthy adults^8^ led to a similar increase of detectable *L. plantarum* in faeces as in the present study. In addition, *L. rhamnosus* 271 has previously been given to healthy adults where it could be recovered in the faeces from 16 of the 17 subjects after 7 days of administration (1.6 × 10^10^ CFU/day), and in three subjects, 7 days after the end of administration^7^. In the present study, *L. rhamnosus* was recovered from all infants in the probiotic group. Lactobacilli were detected in the faeces of 81% of the children at the start of the study, before administration, when most of the children were breastfed and the mean age was about 1.5 months. This is a higher percentage compared to another study in 2-month-old children who were formula-fed with either a normal formula (8%) or a formula supplemented with bovine milk oligosaccharides (BMOS) (17%)^31^. However, adding two strains (*Bifidobacterium longum*, *L. rhamnosus*) to the BMOS formula increased the presence of lactobacilli to 97% after ~1.5 months of the administration, a similar percentage as was detected after 4 and 8 weeks of probiotic administration in the present trial. In the current study, significantly more children were breastfed in the probiotic group than in the placebo group at baseline and this may have affected the results. However, at baseline, the levels and the percentage of infants harbouring the different analysed bacterial groups did not differ between the groups. In addition, comparing the faecal level of the bacterial groups between only breastfed children (*n* = 16 for probiotics, *n* = 11 for placebo) led to the same result; a significant difference was observed between the groups in the levels of *L. plantarum*, *L. rhamnosus* and lactobacilli after 4 and 8 weeks of administration. The other bacterial groups (e.g. *Clostridium* group XI) also changed in a similar way irrespectively if all or only breastfed children were included in the analysis. The number of children harbouring a detectable level of *Clostridium* group XI bacteria was increased over time in both groups, but a lower number of children had detectable levels in the probiotic group than in the placebo group. *Clostridium* group XI bacteria are associated with some harmful effects in humans and one member is *Clostridium difficile* (reclassified as *Clostridioides difficile*)^32^, which is the most common causative agent of antibiotic-associated diarrhoea and colitis^33^.

The microbial diversity did not differ between the probiotic and the placebo group at the end of the administration, but a multivariate analysis showed that the composition of the microbiome was affected after the intake of probiotics. However, further studies are needed to ascertain the bacterial identity of T-RFs changed by probiotic consumption. An earlier study also saw changes in the faecal microbiome in infants given *B. longum* subsp. *infantis* EVC001 from day 7 to day 28 of life, but no significant difference in the Shannon diversity index compared to control^34^. Breast-feeding (especially exclusive breast-feeding) is associated with a low diversity^35^ and analysis of faecal samples at birth, and at 4 and 12 months of age showed that the diversity increased over time, indicating that the microbiome became more and more complex due to cessation of breast-feeding and introduction of other foods^1^. In the present study, most of the children continued to be breastfed (81%) and the median change in diversity after 8 weeks of administration was also minor.

One limitation with the current study is that the intention was to include 20 subjects per group (40 in total) according to ref. ^27^, but the recruitment took longer than planned, and in the end, 36 subjects were included. With this number of subjects, it was possible to evaluate the tolerance of the study product and detect significant differences in the microbiome composition between groups, but further subgroup analyses, for example, to study the microbiome in relation to mode of birth, were not possible. Another limitation was that any changes in safety blood parameters, like the level of d- and l-lactate, were not followed. *Lactiplantibacillus plantarum* HEAL9 produces more d-lactate than l-lactate during fermentation, while *L. rhamnosus* 271 primarily produces l-lactate. It has been speculated that the intake of probiotics can increase the level of d-lactate in the blood of infants, and thus increase the risk of acidosis. However, a recent review of five clinical studies that investigated the level of d-lactate either in the blood or the urine after the intake of probiotics concluded that the intake of probiotics can theoretically increase the level of d-lactate in children <1 year of age, but the increase is subclinical and harmless^36^.

## Conclusion

In conclusion, the probiotic formulation was well tolerated and increased the level of lactobacilli in the intestine. The developed formulation will be used in studies to evaluate additional effects on health with these strains in infants.

## Supplementary information

Supplementary Information

Supplementary Information
