# Supplementary material for: New Lactiplantibacillus plantarum and Lacticaseibacillus rhamnosus strains: well tolerated and improve infant microbiota
Source: Pediatr Res. 2021 Aug 24;91(7):1849–57. doi: 10.1038/s41390-021-01678-1 (PMC9270224; doi:10.1038/s41390-021-01678-1)
Supplement: Supplementary file 2 — Supplementary Information [file 41390_2021_1678_MOESM2_ESM.docx]

**Supplementary Table 1** Primers and probes used for the qPCR analysis of *L. plantarum* and *L. rhamnosus*

| ***Target group*** | ***Primer*** | ***Probe***  ***Dye*** | ***Primer sequence*** | ***Conc (nM)*** | ***Tm(°C)*** |
| --- | --- | --- | --- | --- | --- |
| *L. plantarum* | Forward | - | CGG TGT TCT CGG TTT CAT TAT G | 900 | 58 |
|  | Reverse | - | CCT ACA CAC TCG TCG AAA CTT TGT | 900 | 58 |
|  |  | 6-Fam | CTT GTT CTT TGA AAA CTA G-MGB | 300 | 68 |
| *L. rhamnosus* | Forward |  | ACT GGA TAT CAT TGT TGT AAA | 900 | 59 |
|  | Reverse |  | CGA TGC GAA TTT CTA TTA TTA G | 900 | 59 |
|  |  | 6-Fam | ATT GCC GAG AAC ACA GC-MGB | 300 | 70 |

**Supplementary Table 2** Primers used for the Q-PCR analysis of lactobacilli, *E coli*, bifidobacteria, bacteroides and *Clostridium* group XI (*C difficile*).

| ***Target group*** | ***Primer sequence*** | ***Annealing temp.*** | ***Reference*** |
| --- | --- | --- | --- |
| *E. coli* | Forw: 5´- GAC CTC GGT TTA GTT CAC AGA-3´ | 56°C | Wang et al. 1996 |
|  | Rev: 5´- CCG ACA AAC ACC ACG AAT TCA-3´ |  |  |
| Bifidobacteria | Forw: 5´- TCG CGT C(C/T)G GTG TGA AAG-3´ | 62°C | Rinttilä et al. 2004 |
|  | Rev: 5´- CCA CAT CCA GC(A/G) TCC AC-3´ |  |  |
| *Bacteroides-Prevotella-Porphyromonas* | Forw: 5´- CAC GAA GAA CTC CGA TTG -3´ | 55°C | Hopkins et al. 2005 |
|  | Rev: 5´- CAC TTA AGC CGA CAC CT-3´ |  |  |
| *Clostridium* group XI | Forw: 5´- ACG GTA CTT GAG GAG GA-3´ | 61°C | Song et al. 2004 |
|  | Rev: 5´-GAG CCG TAG CCT TTC ACT-3´ |  |  |
| Lactobacilli | Forw: 5´-AGC AGT AGG GAA TCT TCC A-3´ | 62°C | Rinttilä et al. 2004 |
|  | Rev: 5´-CAC CGC TAC ACA TGG AG-5´ |  |  |

**Supplementary Figure 1.** Multivariate analysis with orthogonal partial least squares to latent structures (OPLS-DA) of samples in the probiotic group. A) OPLS-DA score plot demonstrates the difference between samples before (●) and after (▲) 8 weeks administration of probiotics. B) Predictive loading plot indicates which T-RF increases and decreases after probiotic administration. R2X, 0.167; R2Y, 0.613; Q2, -0.385. t [1], 1st score vector; to [1], orthogonal 1st vector; pq [1], 1st predicted OPLS component (p loading for x and q loading for y); Var ID, variable ID, T-RF size (bp) in this case.

A)

B)
